# Artificial Intelligence Assists in the Detection of Blood Vessels in Whole Slide Images: Practical Benefits for Oncological Pathology

**DOI:** 10.3390/biom13091327

**Published:** 2023-08-29

**Authors:** Anna Timakova, Vladislav Ananev, Alexey Fayzullin, Vladimir Makarov, Elena Ivanova, Anatoly Shekhter, Peter Timashev

**Affiliations:** 1 Institute for Regenerative Medicine, Sechenov First Moscow State Medical University (Sechenov University), 8-2 Trubetskaya St., 119991 Moscow, Russia; timakova_a_a@staff.sechenov.ru (A.T.); fayzullin_a_l@staff.sechenov.ru (A.F.); ivanova_e_i_1@staff.sechenov.ru (E.I.); timashev_p_s@staff.sechenov.ru (P.T.); 2 Medical Informatics Laboratory, Yaroslav-the-Wise Novgorod State University, 41 B. St. Petersburgskaya, 173003 Veliky Novgorod, Russia; vladislav.ananev@novsu.ru (V.A.); vladimir.makarov@novsu.ru (V.M.); 3 B.V. Petrovsky Russian Research Center of Surgery, 2 Abrikosovskiy Lane, 119991 Moscow, Russia; 4 World-Class Research Center “Digital Biodesign and Personalized Healthcare”, Sechenov First Moscow State Medical University (Sechenov University), 8-2 Trubetskaya St., 119991 Moscow, Russia

**Keywords:** digital pathology, deep learning, artificial intelligence, cancer, blood vessel detection

## Abstract

The analysis of the microvasculature and the assessment of angiogenesis have significant prognostic value in various diseases, including cancer. The search for invasion into the blood and lymphatic vessels and the assessment of angiogenesis are important aspects of oncological diagnosis. These features determine the prognosis and aggressiveness of the tumor. Traditional manual evaluation methods are time consuming and subject to inter-observer variability. Blood vessel detection is a perfect task for artificial intelligence, which is capable of rapid analyzing thousands of tissue structures in whole slide images. The development of computer vision solutions requires the segmentation of tissue regions, the extraction of features and the training of machine learning models. In this review, we focus on the methodologies employed by researchers to identify blood vessels and vascular invasion across a range of tumor localizations, including breast, lung, colon, brain, renal, pancreatic, gastric and oral cavity cancers. Contemporary models herald a new era of computational pathology in morphological diagnostics.

## 1. Introduction

Histopathological examination of tissue samples, particularly histological slides, is a critical component of diagnosing and understanding the biological behavior of various malignancies. One of the most significant aspects of histopathological analysis is the evaluation of blood vessels, which play a crucial role in tumor growth, metastasis and response to treatment [1]. Blood vessels within and surrounding the tumor microenvironment contribute to the delivery of essential nutrients and oxygen, facilitating tumor growth and progression. Furthermore, they serve as a route for cancer cells to disseminate from the primary tumor to other sites in the body, leading to metastasis [1,2].

Vascular invasion is indicated as an important prognostic factor in the TNM 8 classification and is present in the College of American Pathologists (CAP) cancer protocol templates [3,4]. The accurate detection and characterization of blood vessels in histological slides is essential for determining tumor stage, predicting prognosis and guiding treatment decisions. One of the commonly used scoring methods is microvessel density (MVD), which measures the number of blood vessels in each area of the tumor. This can be achieved by staining the tissue sample for markers that are specific to blood vessels, such as CD31 or CD34, and then counting the number of stained areas under a microscope [1,2]. High MVD generally indicates a high level of angiogenesis and is often associated with more aggressive tumors and poorer prognosis. Methods include counting vessels in ‘hotspots’ under the microscope or quantifying the expression of angiogenic factors such as VEGF (Vascular Endothelial Growth Factor) by immunohistochemistry (IHC). Some anticancer therapies target angiogenesis, aiming to cut off the tumor’s blood supply and starve it of nutrients [4]. The use of IHC markers of the vascular wall makes the process of searching for blood vessels more visual and faster in high-workflow conditions, reducing the number of errors, while also having disadvantages in the form of the high cost of consumables, a long wait time for staining results, and additional skill requirements for laboratory assistants. Blood vessels possess significant prognostic and predictive value, defined by their role in tumor growth and metastasis.

The presence of lymphovascular invasion (LVI), which refers to the infiltration of tumor cells into the lymphatic or blood vessels, is considered an adverse prognostic factor in many malignancies, including breast, lung and gastrointestinal cancers [5]. LVI has been associated with higher rates of lymph node metastasis, increased risk of distant metastasis and poorer overall survival. The search for lymphatic vessels on a stained hematoxylin and eosin (H&E) preparation is more difficult than the search for blood vessels due to their extremely thin walls and the absence of specific IHC markers, but it is extremely important, for example, in breast cancer to assess the risk of lymphatic metastases.

**Figure 1:**
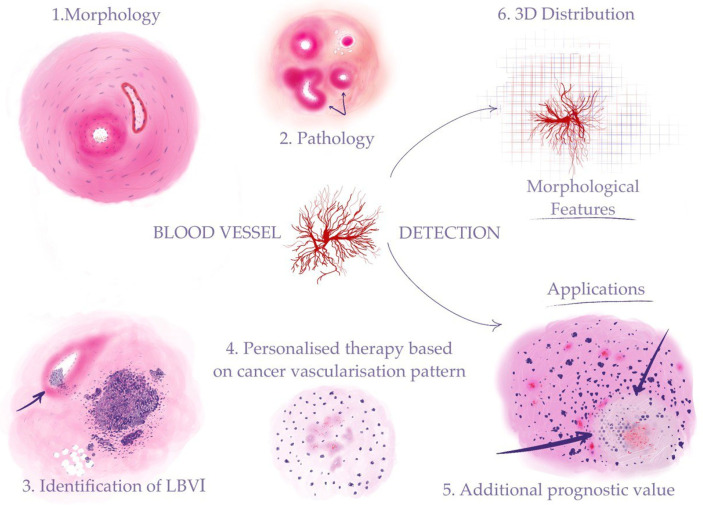
Automated detection of blood vessels makes it possible to investigate a range of morphological features to address clinical problems: (**1**) recognizing morphological structures and patterns (“Morphology” in the figure stands for blood vessels—arteries and veins); (**2**) identifying pathological changes in blood vessels (“Pathology” in the figure stands for sclerosis (indicated by arrows)); (**3**) detection of cancer vascular invasion (the arrow shows tumor cells in the vessel lumen); (**4**) assessment of the tumor vascularization pattern for the purpose of personalized therapy (“Personalized therapy based on cancer vascularization pattern” stands for microvessel proliferation in the glioblastoma); (**5**) AI-assisted calculation of prognostic histological biomarkers (“Additional prognostic value” stands for glioblastoma tumor cells palisading (indicated by arrows) around a central necrosis); (**6**) 3D reconstruction on the cellular level for investigation of tumor growth and its connection to the blood vessel system.

Despite the importance of blood vessel detection and characterization in histological slides, the manual evaluation of these features by pathologists is a time-consuming and labor-intensive process, often prone to inter-observer variability and subjectivity. Moreover, the assessment of blood vessels in histological slides can be challenging due to their complex and heterogeneous morphology, as well as the presence of artifacts and other confounding factors [4]. Automated search for blood vessels and regions of vascular invasion of tumors can provide a fast and reliable solution for optimization of the routine work (Figure 1).

Artificial intelligence (AI) and deep learning techniques, such as convolutional neural networks (CNNs), have emerged as promising tools for automating the analysis of histological slides, including blood vessel detection. These methods have the potential to overcome the limitations of manual evaluation, providing more accurate and consistent results while reducing the workload for pathologists [1,4]. Furthermore, AI-based algorithms can be trained to recognize and quantify various blood vessel features, such as density, morphology, and spatial distribution, which can be challenging for human observers to assess consistently [5].

Several studies have demonstrated the feasibility and effectiveness of AI-based methods for blood vessel detection in histological slides of various malignancies, such as breast cancer, lung adenocarcinoma and oral squamous cell carcinoma. These methods have shown potential in improving the accuracy of LVI detection, predicting lymph node metastasis, and identifying novel morphometric features with prognostic value [2,6]. However, the implementation of AI-based blood vessel detection in clinical practice still faces several challenges, including the need for larger and more diverse datasets, the optimization of algorithms for better performance and clinical utility, and the integration of AI-generated results with existing pathological workflows [2,5,7].

The objective of this review is to provide a comprehensive and up-to-date synthesis of the current literature on AI-based blood vessel detection methods in histological slides, offering valuable insights for researchers, clinicians and decision makers in the field of pathology and oncology. The review will discuss the challenges associated with the implementation of these methods, including data annotation and model interpretability. This review identifies the research gaps and future directions for the development and refinement of AI-based methods, ultimately contributing to the improvement of diagnostic accuracy, prognostic assessment, and personalized treatment strategies in oncology [3,7].

## 2. Automated Blood Vessel Detection in Cancer

Whole slide images (WSI) of cancer tissues allow integration of AI models trained to detect blood vessels (Table 1). In general, segmentation of blood vessels in borders of their walls is helpful for detecting LVI areas and characterizing angiogenesis associated with tumor progression [8]. 

### 2.1. Breast Cancer

Breast cancer is considered the leading cause of death worldwide among women. The incidence of commonly diagnosed cancers worldwide is 2.26 million cases, or 11.7% [35]. The accurate detection and analysis of morphometric features in lymphatic and blood vessel invasion (LBVI) are essential for understanding the progression of breast cancer and predicting lymph node metastases (LNM) [3,4]. A reliable sign of the vascular invasion of cancer is the presence of tumor cells in the lumen of the vessel. Currently, pathologists, when assessing the presence of vascular invasion of breast cancer cells, adhere to a clear distinction between lymphatic and blood vessels, since invasion in the former represents an increased risk of LNM, and in the latter a worse prognosis for survival and the risk of distant metastases [9,36,37]. Invasion is assessed both on the basis of biopsy specimens and in the surgical material, where the presence of invasion is conditionally designated as LBVI1, and the absence of its reliable signs as LBVI0 [3,4,9].

The morphometric features of LVI can provide important prognostic information that may not be readily apparent through manual assessment [6,38,39]. The expert experience embedded knowledge transfer learning (EEKT) model enables the extraction of these features by segmenting LBVIs in breast cancer histopathological images. The predictive value of morphometric features in LNM also extends to the location of LBVI within the tumor [9]. Peritumoral and intratumoral LBVIs have been found to exhibit distinct morphological characteristics, with peritumoral LBVIs being more commonly associated with cancer cell dissemination [9]. The EEKT model can quantify the location of LBVIs by calculating the distance between the LBVI center and the tissue center and margin. This information can provide further insight into the biological behavior of breast cancer and the probability of LNM.

In a different approach, the problem was solved as a direct detection of pathological LVI regions in whole slide images. The model segmented blood vessels with the presence of tumor cells in lumens lined with endothelial cells. The described model demonstrated the ability to predict the occurrence of LNM in a group of LBVI-positive patients. Currently, the presence of LBVI has been demonstrated to possess high prognostic value in LNM prediction, and is routinely evaluated in combination with category T in TNM8, Ki-67 index, histologic grade (G) and immunophenotype. The model made it possible to obtain additional prognostic value from the shape features of LBVI areas: solidity, short-to-long-side ratio of the minimum rectangle, and LBVI-to- minimum-rectangle-area ratio [9]. Another finding of the study was that the count of the LBVI areas did not have the same predictive value as their morphology [9]. The use of this model means for the pathologist a quick and accurate search for LVI, helps to increase the efficiency of the work, and reduces the number of errors in the search for an important factor for predicting the disease [39]. For the patient, this means the selection of more personalized effective tactics for further observation and treatment [20].

### 2.2. Lung Adenocarcinoma

**Figure 2:**
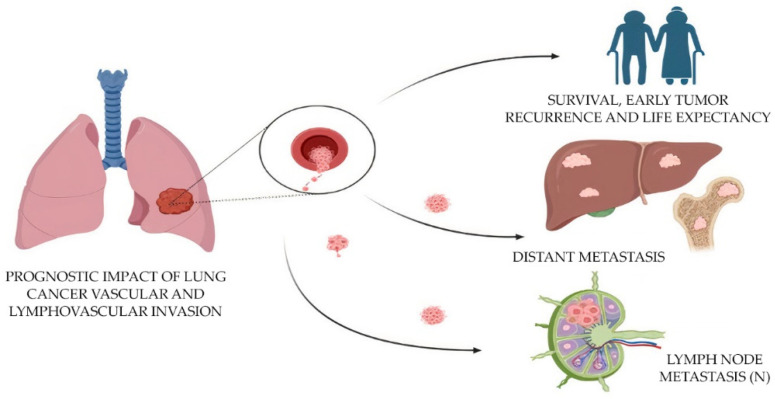
Prognostic impact of the evaluation of lymphovascular invasion in lung cancer.

The combined mortality caused by lung cancer is higher than for any other tumor (worldwide statistics: 1.79 million deaths; 18% incidence of death) [35]. In the case of lung cancer, including adenocarcinoma, there is no methodological separation of tumor invasion into lymphatic and blood vessels [40]. The assessment of vascular invasion (LV1 in the presence of invasion or LV0 in the absence of it) and microvascular density (MVD) in the tumor is important for the prediction of tumor metastasis and survival in histologic specimens [3,4]. To speed up the workflow and improve the accuracy of diagnosis, it is necessary to automate the search for vessels with the presence of tumor invasion, as well as the calculation of MVD. The application of AI and deep learning techniques has shown great potential in the analysis of histopathological images for various malignancies, including lung adenocarcinoma [10,11]. One such approach is the use of CNNs for microvessel detection in hematoxylin and eosin (H&E)-stained images of lung adenocarcinoma tissue [11]. This automated method can provide valuable insights into tumor angiogenesis, which plays a critical role in tumor growth, progression and metastasis (Figure 2).

The use of fully CNNs for microvessel detection has demonstrated promising results. Yi et al. [10] developed a fully CNN that could accurately detect microvessels in H&E images of lung adenocarcinoma. The model was trained on a dataset of manually annotated images and was able to generalize well to new, unseen data. By detecting and quantifying the microvessels within the tumor tissue, the model provided valuable information on the tumor’s angiogenic activity, which has been linked to prognosis and treatment response.

The model was first trained on whole slide images from the TCGA database, and then the model was tested on micropreparation images of patients from the CHCAMS cohort. Segmentation masks were obtained, in which mainly the lumen of the vessel was captured, often with blood cells, sometimes leading to incorrect results and the identification of all structures with erythrocytes in the lumen (mostly, alveolas) as vessels. To increase the accuracy of the neural network, the algorithm was subsequently tested on micropreparation whole slide images that had additional IHC staining for CD34 (vascular endothelial marker) [10].

Despite these advantages, there are some limitations and challenges associated with the use of fully CNNs for microvessel detection in lung adenocarcinoma [10]. One key challenge is the inherent heterogeneity of tumor-associated microvessels, which can exhibit a wide range of cellular origins and morphological characteristics. This can make it difficult for the CNN to accurately differentiate between true microvessels and other similar structures, such as lymphatic vessels or stromal clefts [10]. To address this issue, further research is needed to improve the model’s ability to recognize and distinguish between different types of vessels and to develop more robust training datasets that capture the full spectrum of microvessel morphologies [10].

The fully CNN can be easily adapted and fine-tuned for the analysis of other malignancies or histopathological features. By leveraging transfer learning techniques, the pre-trained model can be fine-tuned on new datasets with minimal additional training, making it a versatile tool for the analysis of various cancer types and histopathological markers [10].

The described algorithm can be used in routine practice to automatically calculate angiogenesis in a tumor. The problem of recognizing “false structures” can be solved through a larger number of trainings with the presence of micropreparations of vessels of various calibers in the whole slide images [10].

### 2.3. Oral Squamous Cell Carcinoma

Oral squamous cell carcinoma (OSCC) is a prevalent and aggressive malignancy with a complex microvascular network [6]. The total incidence is 2%, and the total percentage of deaths is 1.8% [35]. Hypoxia is commonly seen in many solid tumors, including OSCC, due to rapid tumor growth that outstrips the supply of oxygen from existing blood vessels. Hypoxia can influence the behavior of tumor cells and contribute to angiogenesis and metastasis. Tumors often induce angiogenesis in order to supply themselves with the nutrients they need to grow. An increased number of blood vessels in and around the tumor might therefore be indicative of a more aggressive tumor. According to TNM 8 and CAP cancer protocols, lymphovascular invasion (LVI) and tumor angiogenesis have a strong correlation with cancer recurrence, metastasis and poor patient survival [3,4]. The accurate segmentation of microvessels in histological specimens can be considered a preliminary step in the objective identification of LVI and tumor angiogenic analysis [6].

The uncertainty-driven pooling deep learning architecture was applied for the segmentation of microvessels in H&E-stained images of OSCC tissue [6]. This novel approach incorporates uncertainty estimation into the learning process, allowing the model to better adapt to the inherent variability and noise present in histopathological images. This is particularly relevant for microvessel segmentation in OSCC, as the tumor-associated microvessels can exhibit a wide range of morphologies, sizes and staining characteristics, making them challenging to accurately detect and delineate [6]. The trained model was designed to incorporate spatial and morphological information from different pooling scales, enabling it to capture both fine-grained details and larger contextual information. By combining this multi-scale information with an uncertainty estimation mechanism, the model was able to adaptively adjust its predictions based on the local context and the degree of uncertainty in the image. The model achieved high accuracy and consistency, outperforming other state-of-the-art methods in terms of segmentation performance. By providing automated and objective measurements of microvessel density and distribution, the model has the potential to improve prognostic and predictive assessments in OSCC patients [6].

The same research group later reported the FABnet model, which was compared with the most popular neural networks in pathology tasks [12]. The FABnet segmentation model predicted uncertainty maps of nerves and microvessels. The prediction heatmaps obtained by FCN-8, U-Net, Segnet, DeepLabv3+ and the FABnet were shown as overlays on the original images. FABnet demonstrated a precision of 89.35%. The FABnet segmentation mask included only the vessel lumen with blood cell elements, which could limit its efficacy in cases where cancer cells invade the blood vessel wall [12]. The same group of researchers created a more advanced ResNeXt model for the detection of blood vessels and nerves, which showed 99.26% specificity. ResNeXt segmented both vessel lumens and walls, giving it a practical advantage over FABnet [12,13].

### 2.4. Colorectal Cancer

Colorectal cancer (CRC) accounts for more than 1.85 million cases (9.8% of total cancer cases) and causes 850,000 deaths (9.2% of total cancer-related deaths) annually. CRC is the third-most common cause of cancer mortality worldwide [35]. During the colorectal cancer histological specimen examination for predicting tumor metastasis and determining the degree of its aggressiveness, parameters such as vascular density, angiogenesis and LVI are evaluated [3]. In addition, there is an instruction to separate parameters such as budding (tumor buds that are separated from the primary tumor), tumor satellites (these are tumor nests or nodes (macro- or microscopic) that are localized within 2 cm of the primary tumor) and deposits (isolated tumor foci not associated with the primary tumor and lymph node tissue) [3,4]. AI-based methods have been utilized to detect and quantify tumor blood vessels in CRC, and can provide valuable information regarding tumor aggressiveness, metastatic potential and survival prognosis [14]. Both vascular and lymphatic invasion are assessed, denoted by the single abbreviation LV1 [3,14].

A continuous hot spot probability map has been proposed to evaluate whole slide images. First, the preparations are additionally stained for the endothelial marker CD34, and the vessels in the tumor are extracted from the slides by segmentation (“hot spots”). Each such point is taken as a probability. This value gives the probability that an angiogenic hot spot is present at the corresponding location in the original image. The method gives three main results: first, it indicates whether the blood vessels in each tissue sample are randomly distributed or form statistically significant hot spots. Secondly, these hotspots can be accurately located in the image. Thirdly, each point is assigned an exact probability value [14]. Adipose tissue served as a strict control in the study, and no significant angiogenic points could be found in it.

This method has prospective application in vessel segmentation and the statistical comparison of distributions in two- and three-dimensional space, making it possible to obtain a 3D heatmap of microvasculature [14]. Moving from microscopic structures, such as individual small vessels, to angiogenic hot spots of a certain size and distribution, it seems possible to change the measurement scale from micrometers to millimeters. Therefore, histological vascular patterns can be correlated with radiological findings (e.g., tumor perfusion) [14].

### 2.5. Gastric Cancer

The total incidence of gastric cancer is 5.6%, and the total percentage of deaths is 7.7% [35]. In gastric cancer, the depth of invasion is an important prognostic factor that guides the course of treatment. Tumors that have invaded blood vessels may suggest a deeper and more advanced stage of disease. By assessing the involvement of blood vessels in the tumor tissue, pathologists can help determine the depth of invasion of the cancer. Lymphovascular invasion (LVI) is one of the most important prognostic factors in gastric cancer, as it indicates a higher probability of lymph node metastasis and a poorer overall outcome for the patient [15]. Both vascular and lymphatic invasion are assessed, denoted by the single abbreviation LV1 [4,10].

**Figure 3:**
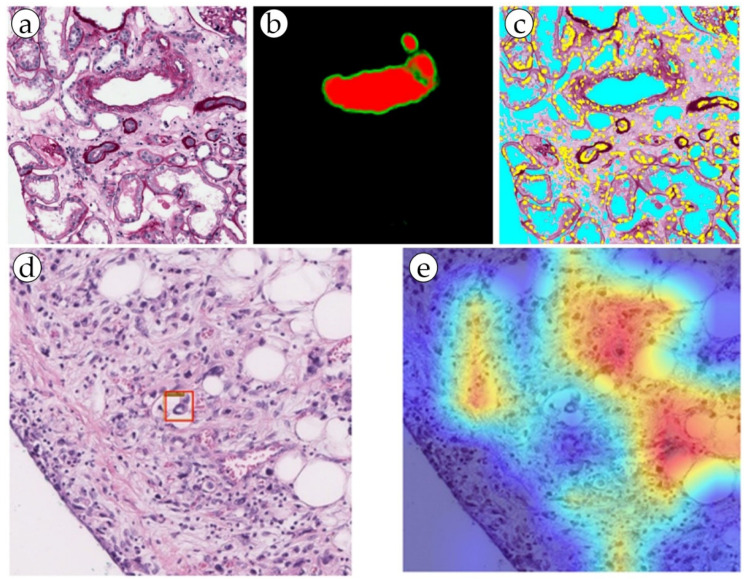
(**a**,**b**) Blood vessel detection by the RENFAST algorithm and (**c**) CNN cellular structure detection (yellow: nuclei; cyan: lumen). Reproduced from [26] under the terms of the Creative Commons CC BY license. (**d**,**e**) Predictive LVI foci (marked with a red box) detection by dual filter (the ConVit and YOLOX models). Reproduced from [15] under the terms of the Creative Commons CC BY license.

The blood vessels were segmented using an AI model for the purpose of LVI detection. The study used 88 whole slide images of histological specimens of gastric adenocarcinoma, which were additionally stained for CD34 and D2-40 vessel markers in a group of LVI+ patients. The ResNet 50, EfficeientNet B3, ConViT (Small) models were fine-tuned on the LVI datasets (Figure 3). For simultaneous localization of LVI regions, a single-stage YOLO object detection model was used, which significantly increased the accuracy of diagnosis. As a result, all LVI(+) patients were identified by the program [15]. This ensemble deep learning model was demonstrated to be robust and accurate, and it can be used as a valuable tool for pathologists in diagnosing gastric cancer and may help improve the accuracy of diagnosis and prognosis of the disease. This approach can be considered an alternative to traditional methods, and as a step toward computer-aided diagnosis systems in histopathology [10,15].

### 2.6. Glioblastoma

Glioblastoma is a highly aggressive brain tumor with poor prognosis. The total incidence of glioblastoma is 1.6%, the total percentage of deaths is 2.5% [35]. A characteristic feature of glioblastoma is pseudopalisading necrosis, which consists of zones of dead cells that appear to line up around areas of vascular necrosis. This pattern is related to the unique way these tumors grow by co-opting the brain’s blood vessels and then causing them to die. Glioblastoma is known for its ability to stimulate angiogenesis. A highly vascularized tumor is characterized by rapid growth and an aggressive course and requires the use of anti-angiogenetic drugs, such as bevacizumab [36]. Glioblastomas often exhibit microvascular proliferation with multiple layers of endothelial cells forming haphazardly arranged, thickened, and distorted vessels. The evaluation of blood vessels, along with other features, helps in grading the tumor, which is important for determining treatment plans and prognostication.

In whole slide images with stains for CD31 and CD34 of glioblastoma histological preparations, microvasculature vessels were detected by creating segmentation masks. Remarkably, the model correctly detected vessels with and without slit-like lumen [16].

AI-based methods have shown the ability to accurately detect microvessels and assess their morphological features in glioblastoma, which can be associated with patient survival and response to therapy [17,18]. In both studies, CNNs were applied to classify glioma and glioblastoma biopsy images according to their grade of malignancy. The trained algorithm reached 96% accuracy, providing reliable support in decision making for pathologists when diagnosing tumor type [17,18]. A more difficult task for artificial intelligence was to determine the cellular subtypes of gliomas: oligodendrocytoma, anaplastic oligodendroma, astrocytoma, anaplastic astrocytoma and glioblastoma. The accuracy achieved was 87%, and the algorithm itself did not contain a mechanism for interpreting the obtained results [17,18]. One of the key benefits of using machine learning in oncological pathology is the integration of different types of data. A neural network was developed for predicting patient survival based on an analysis of the histological and genetic profiles of the tumor. Although the original architecture of the CNN was developed for the program, it has significant potential due to the function of interpreting the results of the analysis [17,18].

### 2.7. Renal Cell Carcinoma

Renal cell carcinoma (RCC) is the most common type of kidney cancer, and it typically manifests as a highly vascular tumor. The total incidence of RCC is 2.2%, and the total percentage of deaths is 1.8% [35]. The presence, shape and size of blood vessels can help in distinguishing between different subtypes of RCC. For example, clear-cell RCC often has a rich network of tiny, thin-walled blood vessels, while papillary RCC might exhibit less vascularization [19].

AI-based methods have been employed to segment and quantify blood vessels in RCC tissue samples, demonstrating their ability to accurately detect microvessels and assess their morphological features. These findings can be associated with tumor stage, grade and patient survival, and can potentially inform treatment decisions and patient management strategies [19]. Leveraging the power of artificial intelligence, the proposed multi-task semi-supervised learning model achieved significant advancements in the area of blood vessel detection. By incorporating both labeled and unlabeled data, this model was able to minimize the dependency on the manual annotation of vascular networks, thereby streamlining the process of automatic segmentation. The model showcased an impressive performance, surpassing that of fully supervised learning models, and exhibited flexibility in its application across various types of tumor, including RCC. The model’s application accuracy for RCC stood at 0.78. The minor loss experienced was likely attributable to the intricate detection of smaller branches of blood vessels and slight variations in blood vessel thickness.

**Figure 4:**
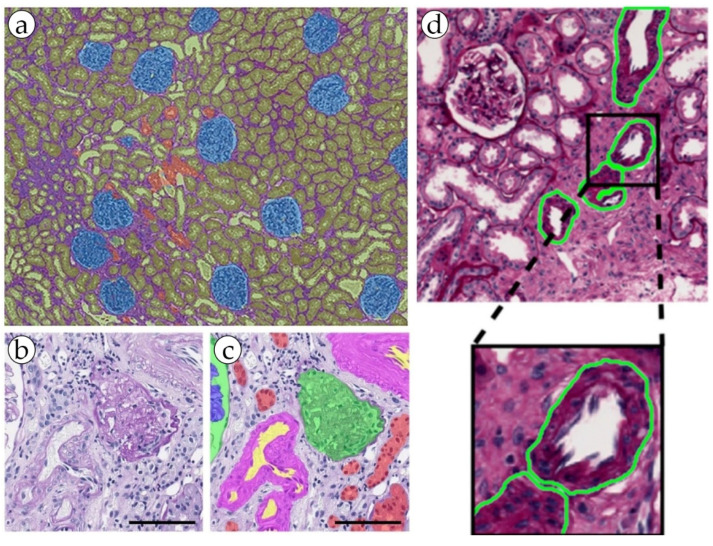
(**a**) An example of kidney structure segmentation by the DeepLab V3+ model. Reproduced from [13] under the terms of the Creative Commons CC BY license. (**b**,**c**) nnU-Net kidney structure segmentation. Reproduced from [22] under the terms of the Creative Commons CC BY license. (**d**) kidney blood vessel detection by the RENFAST algorithm. Reproduced from [26] under the terms of the Creative Commons CC BY license.

The bulk of research in the digital pathology of kidneys that involves the segmentation of blood vessels addresses the problem of non-tumor diseases. It is important to detect blood vessels, distinguish elements such as the endothelium, measure wall thickness and hyalinosis level, and determine the presence or absence of inflammatory cells in a range of kidney pathologies [26,41,42]. These features are strictly necessary in order to perform a diagnosis of acute or chronic transplant rejection, some glomerulo- and vasculopathies, and level of kidney failure in patients with long-term primary of symptomatic hypertension (Figure 3 and Figure 4). In addition, a possible promising task may be the definition of arteritis and the degree of its severity [27,29,30].

### 2.8. Pancreatic Cancer

The total incidence of pancreatic cancer is 2.6%, and the total percentage of deaths is 4.7% [35]. In pancreatic cancer, the invasion of major blood vessels (e.g., the superior mesenteric artery or vein) can be a factor that makes the disease unresectable, meaning it cannot be completely removed by surgery [3,4]. This would typically be associated with a worse prognosis. Specific histologic subtypes of pancreatic cancer may have characteristic patterns of vascular invasion or angiogenesis. Identifying these can help in confirming the diagnosis. An important prognostic marker is the presence or absence of lymphovascular invasion: the tumor spreads along the collagen fibers of the stroma to the membranes of the vessels, and then into their lumen through the bloodstream. There are frequent cases of the detection of tumor cells in the tunica media and tunica adventitia in the absence of tumor emboli.

For a reliable and accurate assessment of vascular invasion of carcinoma in such cases, the CODA model was proposed, which can restore large gaps in ductal and vascular structures in 3D format [31]. The principle of operation of the model is universal and can be applied in the diagnosis of other malignant tumors, such as breast cancer [31,32].

CODA stands for “Cellular Object Detection, Segmentation, and Classification”. It is a framework or methodology used to analyze and interpret digital pathology images with the goal of automating or assisting in the detection, segmentation, and classification of cellular objects within the images. CODA makes it possible to assess the extent of vascular or perineural invasion and precancerous lesions of the pancreas [31]. CODA gives the pathologist a spatial perspective of the course of blood vessels and their branching, and also allows the prediction of the direction of tumor growth into the walls of the blood vessels. Visualization of the bloodstream allows more objective measurements compared to the values of microvessel density. In addition, CODA makes complicated pathological features significantly more understandable, such as leaking vessels [31,32].

It is impossible to adequately stage pancreatic cancer without assessing the blood vessels. Automatic search for regions of lymphovascular invasion and areas of neoangiogenesis will improve the quality of diagnosis, which will undoubtedly have a positive effect on the further treatment of patients [32].

## 3. Machine Learning Approaches to Blood Vessel Detection

**Figure 5:**
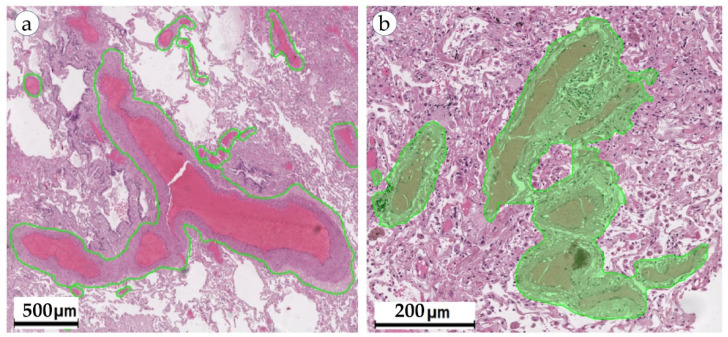
Semantic segmentation of blood vessels, DeepLab V3, WSI of lung cancer, blood vessels selected as green: (**a**) an example of accurate segmentation of blood vessel geometry; (**b**) an example of inaccurate boundary segmentation.

AI models can be applied to extract a range of valuable metrics from images containing blood vessels. For example, information about the exact location and morphology of blood vessels is important for LBVI detection and MVD calculation. When comparing machine learning models, it is convenient to use a formal description of the task of finding vessels in a histological image: “the process of extracting regions of interest relevant to the diagnosed disease”. Semantic segmentation methods based on deep learning are most often used to solve problems related to the selection of areas of interest on WSIs. One of the reasons for this is that when selecting objects on WSIs, it is often necessary to calculate their morphometric parameters. In addition, the shape and size of objects on WSIs often vary greatly (Figure 5a).

**Figure 6:**
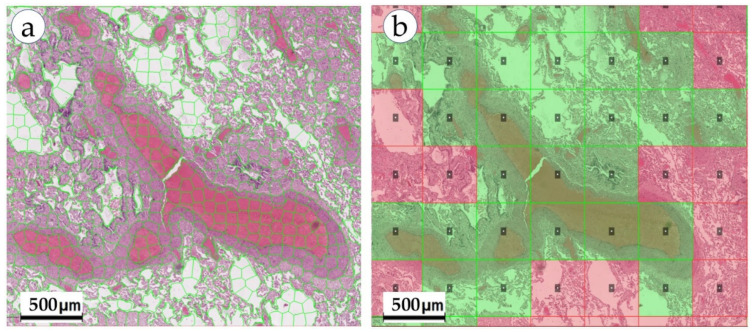
Data representation for various supervised semantic segmentation approaches, DeepLab V3, WSI of lung cancer: (**a**) superpixels; (**b**) patches.

Deep-learning-based semantic segmentation models are highly accurate and can be trained using a small amount of data. With regard to histological images, the essence of semantic segmentation is the assignment of each pixel to a specific tissue class (gland, cell, vessel, etc.). The use of semantic segmentation models assumes the presence of a qualitatively labeled dataset, where each image pixel is assigned to a certain class (Supervised Semantic Segmentation, SSS). For example, a modified U-Net model was used to solve the problem of semantic segmentation of blood vessels and achieved good performance, with 0.89 accuracy and an F1-score of 0.86 [26]. Our review identified the most promising neural network models in the context of solving the problem of semantic segmentation of objects on WSIs:

- DeepLab V3/V3+ [29];
- FPN (Feature Pyramid Network) [10,13];
- HistoSegNet [12,13];
- U-Net/U-Net++/nnU-Net [12,18,20,21,22,24];

With respect to the SSS task, a number of the most popular approaches were distinguished (Figure 6):

- Approaches based on pixel-wise segmentation (classification of each pixel in the image), referred to as the pixel-wise segmentation approach [43];
- Approaches based on the use of superpixels, where a superpixel is a relatively homogeneous group of adjacent pixels (atomic region), referred to as the superpixel segmentation approach [44];
- Approaches based on selecting the center of an object in a sliding window, followed by segmentation of the object’s boundaries, referred to as the patch-wise segmentation approach [45,46];

Pixel-by-pixel segmentation is the most popular approach in the problems of segmentation of tissue structures on WSIs and is able to accurately determine the boundaries of objects in the image. However, the effectiveness of such approaches is largely influenced by factors such as the size and quality of the dataset and the complexity of the model. Since different tissue structures are adjacent to each other and often lack clear visual boundaries, pixel-by-pixel segmentation models are prone to inaccurate segmentation of such areas (for example, incorrect segmentation of the borders of blood vessels, Figure 5b). An alternative approach in such situations is to use superpixels. Compared to traditional pixel approaches, the atomic regions (superpixels, Figure 2b) produced by superpixel generation algorithms represent a natural division of visuals into WSIs. Superpixel-based approaches are often used in combination with CNN models and serve as WSI preprocessing. The use of superpixels allows CNNs to more accurately segment the boundaries of objects, and also increases the computational efficiency of the model. In addition, the effect of superpixel regularization makes it possible to smooth out differences between images obtained from different sources, improving the performance of the model on test data (improving the model generalization ability) [47]. When using patch-wise segmentation approaches, the segmentation result is represented as a rough heatmap (Figure 6b), on which each patch is assigned a binary label indicating the presence/absence of the desired object in the area of this patch. This approach makes it possible to obtain a localization map of the areas of the WSI (i.e., a patch-based heatmap) in which the desired object is supposedly located. However, since this approach does not allow accurate localization of the object boundaries, the results need further processing to refine the object boundaries. For this reason, patch-wise algorithms use WSIs for preliminary analysis to narrow the search area, which in turn allows the efficient use of powerful resource-intensive pixel/superpixel segmentation models.

The data-hungry nature of deep models suggests that in order to obtain a segmentation model with outstanding characteristics, it is necessary to have a large number of images with a high-quality pixel-by-pixel markup. However, due to the large number of problems that arise during the annotation process, for example, due to the low agreement rate of annotators and also due to the high cost of annotation in terms of time and money, it is not always possible to obtain a sufficient number of correctly labeled samples, which, as mentioned earlier, leads to a decrease in the effectiveness of SSS methods [48,49,50]. To solve this problem, image segmentation models have been developed that can be trained with weaker and cheaper labels [51,52]. These studies have led to the emergence of the Weakly Supervised Semantic Segmentation (WSSS) approach [53,54]. WSS is a general term covering a variety of approaches aimed at building predictive models that can be trained on limited datasets. Typically, WSSS is used in the following situations:

- Only a small part of the dataset has been annotated;
- Presence of samples with rough/inaccurate labeling;
- Presence of samples with mistakes in the annotation (incorrect markup).

SSS methods show high accuracy due to their pixel-wise classification, taking into account the presence of a well-labeled dataset [55]. However, SSS models show a significant decrease in predictive performance when trained using incorrectly or roughly labeled data, while the performance of WSS models trained on the same data decreases slightly [51,53]. To solve the LBVI problem, it is necessary to find the boundaries of the blood vessels as accurately as possible. Considering this, as well as the fact that there are no large WSI datasets with blood vessel labeling in the public domain, the development of blood vessel segmentation models that can be translated into practice requires the combination of a range of approaches. For example, a patch-wise segmentation approach can be applied to obtain a heatmap of the areas in which the vessels are located at the first stage for the reduction of computational cost. In the second step, each segment from the WSI heatmap can be segmented using a pixel-/superpixel-wise model to obtain more accurate segmentation masks corresponding to blood vessel morphology. It is recommended that WSSS be used in situations where non-SSS methods do not achieve the required metric or the amount of data labeled by the experts is limited.

## 4. Challenges and Perspectives

Despite the numerous advantages of and advances in AI-based methods for blood vessel detection in histological slides, several challenges remain to be addressed for their effective implementation in clinical practice. This section discusses the key challenges to be faced in implementing these methods, and possible solutions for overcoming them.

(1) Variability in slide preparation and staining techniques can lead to inconsistencies in the appearance of blood vessels, which may negatively impact the performance of AI-based methods. This challenge can be mitigated by developing robust models that can handle such variations, incorporating data augmentation techniques during training, and standardizing slide preparation and staining procedures.
(2) AI-based methods, especially deep learning approaches, require large amounts of annotated data for training [21]. The manual annotation of blood vessels in histological slides is time consuming and prone to inter-observer variability. To address this challenge, researchers can employ active learning strategies to optimize the use of annotated data, develop semi-automated annotation tools to assist pathologists and explore the potential of synthetic or simulated data to augment the training dataset. The generalizability of AI models relies on their ability to perform well across different patient populations, histological slide preparations, and staining techniques. Larger and more diverse datasets ensure that AI algorithms are trained and tested on a wider range of variations, which in turn improves the model’s ability to accurately detect blood vessels in different clinical scenarios [22].
(3) Deep learning models can be difficult to interpret and explain, which can hinder their adoption in clinical practice [23]. To overcome this, researchers should focus on developing explainable AI (XAI) methods that provide insights into the underlying decision-making process of the model [56]. Techniques such as saliency maps, layer-wise relevance propagation and attention mechanisms can help improve the interpretability of AI-based methods for blood vessel detection. For AI-based blood vessel detection methods to be successfully adopted in clinical practice, they must be seamlessly integrated into existing clinical workflows [57].
(4) The implementation of AI-based methods in clinical practice raises ethical and legal concerns, such as data privacy, informed consent, and liability for misdiagnosis. Researchers and healthcare professionals should work together to establish guidelines and policies that address these concerns, ensuring the responsible and ethical use of AI-based methods for blood vessel detection.
(5) One of the major challenges associated with AI models, particularly deep learning methods, is their black-box nature, meaning that the decision-making process of the model is not easily understandable by humans. A human-in-the-loop approach can address this issue by involving clinicians in the model development and validation process. By providing feedback on AI-generated results, clinicians can help improve the transparency and interpretability of the models, ensuring that AI technology is more clinically relevant and applicable [24].

Researchers and clinicians should carefully weigh the advantages and limitations of different AI methods in order to select the most suitable approach for their specific application. Future studies should continue to explore novel AI-based methods that address current limitations and enhance the overall performance and usability of automated blood vessel detection in the histological slides of various malignancies.

## 5. Projections for Clinical Translation

AI-based blood vessel detection can offer insights into the biology and microenvironment of tumors, which can be critical for predicting treatment response. For example, blood vessel characteristics can be used to predict the response to anti-angiogenic therapy or to identify patients who may benefit from specific therapeutic strategies [58]. AI can be used to monitor changes in blood vessel features during treatment, providing early indicators of treatment efficacy and allowing for timely adjustments in therapy.

The predictive power of AI-based blood vessel detection can be further enhanced by integrating it with genomic and proteomic data [28]. Combining these different data types could provide a more comprehensive understanding of the biology and microenvironment of tumors, leading to improved prognostic and predictive models. For example, the integration of AI-based blood vessel features with gene expression profiles can help identify specific molecular subtypes of cancer that could be associated with distinct prognostic and treatment response profiles.

While the focus of this review was on blood vessel detection, it is worth noting that AI-generated morphometric features can be extended to other histological structures, such as immune cell infiltrates and stromal components. The evaluation of the tumor microenvironment may provide additional insights into tumor behavior and prognosis.

## 6. Conclusions

This review provides a comprehensive overview of AI-based solutions for blood vessel detection in whole slide images of histological slides for various malignancies. The studies reviewed have demonstrated the potential of AI algorithms to enhance the accuracy, efficiency, and reproducibility of blood vessel detection, contributing to improved diagnostic and prognostic assessments in oncology. The integration of AI with clinical practice can help pathologists make well-informed decisions, reducing inter-observer variability and improving patient management. Furthermore, the predictive value of AI-generated morphometric features may uncover novel insights into tumor biology and treatment response.

## Figures and Tables

**Table 1:** AI models that have been applied to detect tissue structures including blood vessels in WSI.

No.	Article Title	Cancer Site	AI Model Name and Description	Accuracy	Advantages (+)/ Disadvantages (−)
1	Chen, Y.; et al. Further predictive value of lymphovascular invasion explored via supervised deep learning for lymph node metastases in breast cancer [9]	Breast	EEKT model (based on DeepLab V3+)— object detection model	0.9300	DL (Deep Learning) model showed the ability to quantify LBVI and identify its added predictive value (+) Unable to segment small vessels (−)
2	Yi, F.; et al. Microvessel prediction in H&E stained Pathology Images using fully convolutional neural networks [10]	Lung	FCN and FCN-8 models—object detection model	FCN—0.9520; FCN-8—0.9460	FCN model algorithm may have a false positive problem for background regions where a large number of blood cells appear (−)
3	Vu, Q.D.; et al. Methods for Segmentation and Classification of Digital Microscopy Tissue Images [11]	Lung	ResNet50—object detection model	0.8100	Method primarily focuses on the diagnostic areas within the image for determining the cancer type (+)
4	Fraz, M.M.; et al. FABnet: feature attention-based network for simultaneous segmentation of microvessels and nerves in routine histology images of oral cancer [12]	Oral cavity	FABnet (U-Net, SegNet, DeepLabv3+, FCN-8)— pixel segmentation (heatmaps) plus object detection model	FABnet—0.9705; U-Net—95.18; SegNet—92.34; DeepLabv3+—97.21; FCN-8—94.46	Segments the microvessels and nerves in routinely used H&E-stained images (+) FCN-8, U-Net and DeepLabv3+ are unable to segment small vessels; SegNet merges the two closely located by vessels into one large vessel
5	Fraz, M.M.; et al. Uncertainty Driven Pooling Network for Microvessel Segmentation in Routine Histology Images [6]	Oral cavity	Xception model—the object detection model	0.9694	The proposed method successfully segments small vessels and closely located vessels as different ones (+)
6	Rasool, A.; et al. Multiscale Unified Network for Simultaneous Segmentation of Nerves and Microvessels in Histology Images [13]	Oral cavity	ResNeXt 50, FCN8, U-Net, SegNet, Deeplabv3+—object detection models	ResNeXt 50—0.9785; FCN8—0.9693; U-Net—0.9518; SegNet—0.9234; Deeplabv3+—0.9721	It can generate consistent and more refined shapes of irregular dimensional objects (+)
7	Kather, J.N.; et al. Continuous representation of tumor microvessel density and detection of angiogenic hotspots in histological whole-slide images [14]	Colon	MATLAB—pixel segmentation model (heatmaps)	Not reported	By turning from microscopic structures like single, small vessels to angiogenic hotspots, it seems to be possible to change the measurement scale from μm to mm. Consequently, histological vascular patterns could be compared with radiological data (+)
8	Noh, M.-g.; et al. Ensemble Deep Learning Model to Predict Lymphovascular Invasion in Gastric Cancer [15]	Stomach	YOLOX—object detection models	0.9648	YOLOX model can predict the LVI foci using a bounding box (+) A number of LVI(+) foci imbalances may exist for each slide; LVI(+) foci always contain the possibility of false positives or negatives (−)
9	de Castelbajac, M. Automated segmentation of blood vessels in immuno-stained whole slide images [16]	Brain	Segmentation based on HSV color model and radial algorithm for detecting open vessels (object detection model)	0.8600	Obvious bright and opened vessels are correctly retrieve (+), but not when the lumen is too small or partly stained like on the right (−)
10	Zadeh Shirazi, A.; et al. A deep convolutional neural network for segmentation of whole-slide pathology images identifies novel tumor cell-perivascular niche interactions that are associated with poor survival in glioblastoma [17]	Brain	DCNN—pixel segmentation model	0.8600	The model can segment unclear regions in the original slide (+), can tackle the problem of over-segmentation of the cellular tumor microvascular (+)
11	Li, X.; et al. Microvascularity detection and quantification in glioma: a novel deep-learning-based framework [18]	Brain	GoogLeNet—object detection model	0.9570	The accuracy of microvessel recognition has a large margin of improvement due to the segmentation error and the over counting, especially in larger pathological images with complex content (+)
12	Xiao, R.; et al. Multi-task Semi-supervised Learning for Vascular Network Segmentation and Renal Cell Carcinoma Classification [19]	Kidney	HRNet—patch-wise segmentation approach	0.9369	Model reduces the reliance on manually vascular network masks and achieves automatic segmentation (+). This model can outperform the fully supervised learning model and is versatile in other types of tumors (+)
13	O’Toole, J.; et al. Development and evaluation of deep learning–based segmentation of histologic structures in the kidney cortex with multiple histologic stains [20]	Kidney	U-net—object detection model	0.9705	Model correctly identified small fragments of tunica media despite the lack of a lumen (+)
14	Bouteldja, N.; et al. Deep Learning–Based Segmentation and Quantification in Experimental Kidney Histopathology [21]	Kidney	U-Net—object detection model	0.8810	Multiclass segmentation of renal histology and vascular pathology (+) The nonsegmented area comprises peritubular capillaries, arterial adventitia (−)
15	Klinkhammer, B.M.; et al. Next-Generation Morphometry for pathomics-data mining in histopathology [22]	Kidney	U-Net—multiclass segmentation model	0.8700	Multiclass segmentation of renal histology and vascular pathology (+) Model is unable to segment peritubular capillaries (−)
16	Deng, R.; et al. Omni-Seg: A Scale-aware Dynamic Network for Renal Pathological Image Segmentation [23]	Kidney	Omni-Seg+—object detection model	0.9660	The proposed method achieves superior segmentation performance with less computational resource consumption (+)
17	Hermsen, M.; et al. Hermsen, M.; et al. Deep Learning–Based Histopathologic Assessment of Kidney Tissue [24]	Kidney	U-net—object detection model	0.8900	Unable to segment peritubular capillaries (−)
19	Bevilacqua, V.; et al. An innovative neural network framework to classify blood vessels and tubules based on Haralick features evaluated in histological images of kidney biopsy [25]	Kidney	BPNN—object detection model	0.8920	High accuracy when trained on a limited dataset (+)
20	Salvi, M.; et al. Karpinski Score under Digital Investigation: A Fully Automated Segmentation Algorithm to Identify Vascular and Stromal Injury of Donors’ Kidneys [26]	Kidney	RENFAST (Rapid EvaluatioN of Fibrosis And vesselS Thickness)—multiclass segmentation model	0.9443	Detection of all structures of the blood vessel (+)
21	van der Laak, J.; et al. Deep learning in histopathology: the path to the clinic [27]	Kidney	CPATH (combination of U-Net models)—object detection model	0.9700	High accuracy in detecting arteriols (+)
22	Gadermayr, M.; et al. Segmenting renal whole slide images virtually without training data [28]	Kidney	Polygon-fitting segmentation method	0.8600	Gives an opportunity to segment structures without training data
23	Lee, J.; et al. Unsupervised machine learning for identifying important visual features through bag-of-words using histopathology data from chronic kidney disease [29]	Kidney	DeepLab V3+ with ResNet-18 architecture, pre-t ImageNet—object detection model	0.9500	Can help to discover previously unknown features that are useful for categorizing and predicting patient outcomes without human input (+)
24	Farris, A.B.; et al. Artificial intelligence and algorithmic computational pathology: an introduction with renal allograft examples [30]	Kidney	GoogLeNet—object detection model	0.9500	DL segmentation of arteries, arterioles, and peritubular capillaries (+)
25	Kiemen, A.L.; et al. CODA: quantitative 3D reconstruction of large tissues at cellular resolution [31]	Pancreas	CODA—multiclass segmentation model with vessel 3D-reconstruction	>90%	CODA gives the pathologist a spatial perspective of the course of blood vessels and their branching, and also allows prediction of the direction of tumor growth into the walls of the blood vessels
26	Kiemen, A.L.; et al. Tissue clearing and 3D reconstruction of digitized, serially sectioned slides provide novel insights into pancreatic cancer [32]	Pancreas	CODA—multiclass segmentation model with vessel 3D-reconstruction	>90%	CODA gives the pathologist a spatial perspective of the course of blood vessels and their branching, and also allows prediction of the direction of tumor growth into the walls of the blood vessels
27	Gao, E.; et al. Automatic multi-tissue segmentation in pancreatic pathological images with selected multi-scale attention network [33]	Pancreas	SMANet is based on U-Net with 5 levels—object detection mode	0.7690	Multi-scale attention network is proposed to realize the segmentation of tumor cells, blood vessels, nerves, islets and ducts in pancreatic pathological images (+)
28	Niazi, M.K.K.; et al. Grading vascularity from histopathological images based on traveling salesman distance and vessel size [34]	Bone marrow	MaxLink algorithm—object detection mode	0.6820	Gives the opportunity to associate the grading information with the patient outcome (+)

## Data Availability

Not applicable.
